# A WIMSical approach to decoding DNA methylation in myeloid leukemia

**DOI:** 10.1186/s13059-014-0441-z

**Published:** 2014-09-02

**Authors:** Olga A Guryanova, Ross L Levine

**Affiliations:** Human Oncology and Pathogenesis Program, Memorial Sloan Kettering Cancer Center, New York, NY 10065 USA; Leukemia Service, Department of Medicine, Memorial Sloan Kettering Cancer Center, New York, NY 10065 USA

## Abstract

Integrated transcriptomic and high-resolution whole genome methylation analysis in a myeloid leukemia cell line defines genes that respond to clinically relevant DNA methyltransferase inhibitors.

Nucleotide sequence serves as the essential text of the genetic ‘book’ known as the human genome, but this book also comes with ‘reading instructions’, ‘bookmarks’ and ‘highlighted passages’ in the form of epigenetic patterns. Not surprisingly, the initiation and progression of cancer are governed not only by the accumulation of genetic lesions in the text itself but also by perturbations in the epigenome. Indeed, epigenetic alterations that dysregulate the ability of cancer cells to utilize genomic information are increasingly being appreciated as an important contributing factor in the development of different malignancies [1].

## Epigenetic therapies for malignancies

Unlike genetic lesions, epigenetic patterns are relatively fluid and might be correctable by therapeutic perturbations. Such malleability has made epigenetic therapies an attractive alternative to cytotoxic chemotherapies. Rather than trying to destroy rapidly dividing cells instantly, epigenetic therapies aim to reprogram cancer cells by attenuating cancer-promoting transcriptional networks [1]. These therapies, specifically the DNA hypomethylating agents 5-azacitidine (Aza) and 5-aza-2′-deoxycytidine (decitabine, Dac), have been used increasingly for the treatment of patients with myelodysplastic syndrome (MDS) and acute myeloid leukemia (AML) [2]. Despite the successful use of both Aza and Dac in the clinic, the molecular mechanisms behind their efficacy requires additional investigation. In this issue of *Genome Biology*, Lund *et al*. [3] employ a sophisticated methodology, which they term WIMSi, to sift through high-resolution DNA methylomic and transcriptomic data in an attempt to identify the gene targets of hypomethylating therapies [3].

Myelodysplastic syndrome (MDS) and acute myeloid leukemia (AML) are malignant disorders of hematopoietic progenitor cells that lead to defective hematopoiesis. MDS is characterized by cytopenia (a reduction in the number of blood cells); AML, on the other hand, is typified by leukocytosis, which primarily results from the expansion of immature myeloid blasts. Both types of malignancies are most commonly diagnosed later in life (median age at AML diagnosis is 68 years). An intense interest in demethylating therapies for AML and MDS has been fueled by the presence of hallmark focal DNA hypermethylation that distinguishes leukemic blasts from normal hematopoietic progenitors, and by the high frequency of somatic mutations in genes that regulate the methylation of genomic DNA, including *IDH1/2*, *TET2*, and *DNMT3A* [1,2,4,5], in myeloid malignancies.

## Under investigation: mechanism of action of hypomethylating drugs

The classical view of the establishment and maintenance of site-specific DNA methylation posits that methyl groups are introduced at CpG palindromic sites by *de novo* DNA methyltransferases (DNMTs; DNMT3A and DNMT3B) over the course of cellular differentiation, starting in embryogenesis. After each round of replication, the methylation pattern can be copied from the (methylated) parent strand to the (unmethylated) daughter strand by the maintenance methyltransferase DNMT1 because of its preference for hemimethylated DNA. The TET family of enzymes can mediate active DNA demethylation, although the steps subsequent to TET enzymatic activity have not been fully elucidated. In replicating cells, DNA methylation also decreases passively in the absence of re-methylation [1]. Passive demethylation is thought to underlie the mechanism of action of DNMT inhibitors. The nucleoside analogs Aza and Dac, and their metabolites, act as DNMT suicide substrates upon incorporation into replicating DNA. Additionally, exposure to higher doses of these agents results in bulky adduct formation and DNA damage [2,6].

Cancer-associated focal DNA hypermethylation, especially in the promoter regions of key effector genes, is believed to be responsible for silencing critical tumor suppressors, such as pro-apoptotic genes and regulators of the cell cycle and differentiation commitment [1,6,7]. Treatment with DNMT inhibitors induces early loss of DNA methylation, even at low drug doses [6,8], but published studies report discordant effects of hypomethylating agents on gene expression [6-8]. Some investigators observed widespread reactivation of silenced tumor suppressor genes in response to DNMT inhibitors. Others failed to detect any relationship between DNA hypomethylation and gene expression, despite consistent robust correlation between DNA methylation and gene repression in untreated primary cultured cells and cell lines [6,7,9]. The very short extent of treatment (most studies chose 48 to 72 hour timepoints) [6,8] may not be sufficient to induce robust changes in gene expression despite early widespread hypomethylation. In addition, the accumulation of DNA methylation during gene silencing precedes the deposition of repressive histone marks and chromatin compaction. It is conceivable that the same would hold true during gene reactivation and would require days rather than hours.

As an alternative hypothesis to explain why there was no correlation between gene expression and DNA methylation changes, Lund *et al*. [3] reasoned that the limited resolution and skewed representation of genomic features inherent in a previous generation of experimental methods were to blame. To zero in on a list of candidate genes regulated exclusively by DNA methylation, Lund *et al*. [3] investigated a combined dataset of high-resolution whole-genome bisulfite sequencing (WGBS) data and gene expression profiles by RNA-seq in an AML cell line treated with Aza. Although higher than that used in previously published studies, a low dose of Aza (0.5 μM) was chosen to minimize changes in cell proliferation and DNA damage. DNA damage and changes in proliferation rates induced by high-dose Aza may obscure the impact of DNA demethylation on gene expression. To avoid this the authors chose a significantly lower dose that did not affect proliferation and did not induce DNA damage.

The study focused on the OCI/AML-3 leukemic cell line, which is known to carry mutations in the *NPM1* and *DNMT3A* genes. The nucleophosmin gene *NPM1* is one of the most frequently mutated genes in AML. Mutations in NPM1 most often disrupt its nuclear localization signal and are associated with favorable prognosis [4,5]. Even though NPM1 is not directly involved in the regulation of DNA methylation, mutated NPM1 defines a distinct epigenetic cluster identified by the DNA methylation profiling of 344 AML patients [9]. *DNMT3A* is another gene that is frequently mutated in AML. In contrast to *NPM1* mutations, *DNMT3A* mutations predict poor survival rates [1,5]. The presence of two mutations with opposite prognostic effects challenges unequivocal risk stratification. Despite the frequent occurrence of mutations in both *NPM1* and *DNMT3A* in AML, the co-occurrence of mutations in both of these genes in the absence of *FLT3* internal tandem duplications (FLT3-ITD) is relatively uncommon [4]. Consequently, the OCI/AML-3 cell line does not reflect the genotypic context most commonly observed in AML patients whose leukemia cells carry these mutant disease alleles.

DNMT3A is directly involved in the establishment and maintenance of DNA methylation patterns, and *DNMT3A* mutant AML patient-derived samples show a small but significant decrease in mean methylation relative to non-mutated samples [10]. Despite having solid adverse prognostic implications, the predictive value of *DNMT3A* mutations with respect to the response to hypomethylating agents has not been conclusively established. Overall, four small-scale clinical trials that investigated the relationship between *DNMT3A* mutations and response to hypomethylating agents showed a trend towards prolonged progression-free survival (PFS) and higher complete remission (CR) rate in those carrying the mutation [2].

The authors’ general characterization of the DNA-methylation landscape by WGBS confirmed previous findings [6,7,9]: methylation is enhanced in the bodies (transcribed portions) of highly expressed genes, but there are low methylation levels in promoters and an almost complete loss of methylation just downstream of transcriptional start sites. Treatment of OCI/AML-3 cells with Aza resulted in general, genome-wide hypomethylation; genomic features with the highest levels of methylation pre-treatment were affected to the greatest extent. Consistent with most previously published studies, there was no obvious correlation between DNA hypomethylation at gene promoters or CpG islands and expression of the corresponding gene. The authors then used WIMSi (Washington University Interpolated Methylation Signatures), a more sophisticated computational approach, to tease out subtle co-regulation between DNA methylation and gene expression [3].

## WIMSi analysis identifies genes directly regulated by DNA methylation

WIMSi analysis relies on the identification of gene clusters that share similar DNA methylation profiles by shape-curve and concordant changes in gene expression before and after treatment with Aza to identify specific genes or loci of interest. This approach yielded a list of 246 derepressed genes that have functions in cell death and survival or in cell movement and proliferation, all presumably regulated directly by DNA methylation. Cross-validation with other published datasets showed that WIMSi genes were enriched among genes whose expression was downregulated in AML compared to normal hematopoietic stem and progenitor cells (HSPCs). Many WIMSi genes were also found as upregulated in a previous study investigating the impact of DNMT inhibitors on methylation and gene expression in primary AML samples that failed to detect a relationship between DNA hypomethylation and gene expression by more general methods [6]. Overall, the study by Lund *et al*. [3] demonstrates the feasibility of using high-resolution WGBS and RNA-seq, when combined with sophisticated computational methods, to uncover relationships between DNA methylation and gene expression.

As with any novel methodology, this study raises many questions for subsequent investigation. First, the question of whether other epigenetic marks are involved in early regulation of gene expression is the most important and the most informative. Are DNA methylation and histone modifications co-regulated, or does one direct the other? What is the timing of the chromatin reorganization that is necessary to allow gene de-repression, and what is the optimal time point at which to measure it? Would histone modifications be more directly related to changes in gene expression than changes in DNA methylation? Comprehensive epigenetic profiling that integrates analysis of the DNA methylome, the epigenome and the transcriptome will further our understanding of the mechanisms behind the efficacy of hypomethylating therapies. Such studies will be welcomed with great enthusiasm in the field of clinical cancer epigenetics. Second, what are the implications of the apparent reprogramming of OCI/AML-3 cells into a state more similar to HSPCs after Aza exposure? Are the effects on self-renewal genes distinct from those on differentiation and lineage-commitment genes? How similar are the reprogrammed profiles to those of more mature populations? Detailed studies involving additional leukemic and normal samples will be needed to shed light on the complex processes that guide epigenetic reprogramming. Finally, how generalizable are these findings, given the specific genetic make-up of the OCI/AML-3 cells and the uncertain impact of *DNMT3A* mutational status on predicting response to hypomethylating agents? Extension of these studies to cellular models (cell lines or primary AML samples) with more relevant and comprehensive mutational landscapes will be eagerly awaited.

In the book of life that is the genome, aberrant DNA hypermethylation that silences tumor suppressor genes can be likened to pages that are stuck together, concealing important plot twists. Hypomethylating therapies can peel these pages apart and open the book at the right chapter. Whether it will be fully readable and able to guide the reprogramming of malignant cells, and what other signals or epigenetic marks are needed to promote proper genome reading, are questions that we are just beginning to answer.

## Abbreviations

AML: Acute myeloid leukemia
Aza: 5-azacitidine
CR: Complete remission
Dac: 5-aza-2′-deoxycytidine
DNMT: DNA methyltransferase
FLT3-ITD: FLT3 internal tandem duplication
HSPC: Hematopoietic stem and progenitor cells
MDS: Myelodysplastic syndrome
PFS: Progression-free survival
WGBS: Whole genome bisulfite sequencing.
